# Comparison of the different anti-CD16 antibody clones in the activation and expansion of peripheral blood NK cells

**DOI:** 10.1038/s41598-023-36200-6

**Published:** 2023-06-11

**Authors:** Jinho Kim, Minh‑Trang Thi Phan, Ilwoong Hwang, Jeehun Park, Duck Cho

**Affiliations:** 1 Department of Health Sciences and Technology, SAIHST, Sungkyunkwan University, Seoul, Korea; 2 Falcuty of Applied Technology, School of Technology, Van Lang University, Ho Chi Minh City, Vietnam; 3 Cellgentek Corp, Cheonju, Korea; 4 Soft Foundry Institute, Seoul National University, Seoul, Korea; 5 Department of Laboratory Medicine and Genetics, Samsung Medical Center, Sungkyunkwan University School of Medicine, 81, Irwon-Ro, Gangnam-Gu, Seoul, 06351 South Korea; 6 Cell and Gene Therapy Institute (CGTI), Samsung Medical Center, Seoul, Korea

**Keywords:** Biological techniques, Immunology

## Abstract

Natural killer (NK) cells are promising tool for cancer treatment. Methods have been developed for large-scale NK cell expansion, including feeder cell-based methods or methods involving stimulation with NK cell activating signals, such as anti-CD16 antibodies. Different clones of anti-CD16 antibodies are available; however, a comprehensive comparison of their differential effects on inducing NK cell activation and expansion has not been conducted among these various clones under the same experimental conditions. Herein, we found that the NK cell expansion rate differed depending on the various anti-CD16 antibodies (CB16, 3G8, B73.1, and MEM-154) coated on microbeads when stimulated with genetically engineered feeder cells, K562‑membrane-bound IL‑18, and mbIL‑21 (K562‑mbIL‑18/-21). Only the CB16 clone combination caused enhanced NK cell expansion over K562‑mbIL‑18/-21 stimulation alone with similar NK cell functionality. Treatment with the CB16 clone once on the initial day of NK cell expansion was sufficient to maximize the combination effect. Overall, we developed a more enhanced NK expansion system by merging a feeder to effectively stimulate CD16 with the CB16 clone.

## Introduction

Natural killer (NK) cells are highly cytotoxic and are considered a component of innate immunity against infections and tumors^1–3^. NK cells constitute approximately 10–15% of the lymphocytes in humans; most NK cells have the morphology of large granular lymphocytes and are usually defined as CD3− CD56+ cells^4–6^. Owing to their high cytotoxic activity against cancer cells, adoptive transfer of NK cells is a promising therapeutic strategy; therefore, obtaining large numbers of activated NK cells is important for effective NK cell-based immunotherapy.

Multiple approaches have been used to expand NK cells, such as the use of cytokines (IL-2, IL-15, IL-21, and IL-18) or feeder cell lines. Feeder-free methods overcome the risk of viable feeder cell proliferation and contamination, but in general results in a lower fold expansion. However, feeder cell lines provide remarkable NK cell expansion and improved NK cell activity, compared to methods using only cytokines^7–9^. Therefore, clinical scale examples of feeder cell line based expansion from primary NK cells have been reported, including γ-irradiated Epstein-Barr virus transformed lymphoblastoid cell line, K562 cell line, and genetically engineered (GE) K562.mbIL21.4-1BBL feeder cells)^10^. Recently, GE feeder cells express cytokines (i.e., mbIL18) and costimulatory factors (i.e., OX40L, and anti-CD16 antibody) that have been extensively studied for more effective NK cell expansion^11–14^.

CD16 (Fcγ receptor III) is a receptor expressed on NK cells that facilitates antibody-dependent cellular cytotoxicity (ADCC) by binding to the Fc portion of various antibodies. The enhancement of NK cell activity upon interaction of cells with anti-CD16 antibodies has been reported^15^. CD16 ligation also affects NK cell proliferation by enhancing the entry of NK cells into division^16^. Although several clones of anti-CD16 antibodies have been used in some studies as an effective method to expand NK cells^17,18^, a direct comparison of anti-CD16 Ab clones for the expansion and activation of primary NK cells has not been reported.

In this study, we evaluated the differences in NK cell response and expansion of four anti-CD16 antibody clones, namely CB16, 3G8, B73.1, and MEM-154, in the same NK cell expansion system. Magnetic microbeads coated with anti-CD16 antibody and the genetically engineered feeder cells, K562‑mbIL‑18/-21, were used to stimulate the NK cells.

## Result

### NK cell responses upon stimulation with different anti-CD16 antibody clones

**Figure 1:**
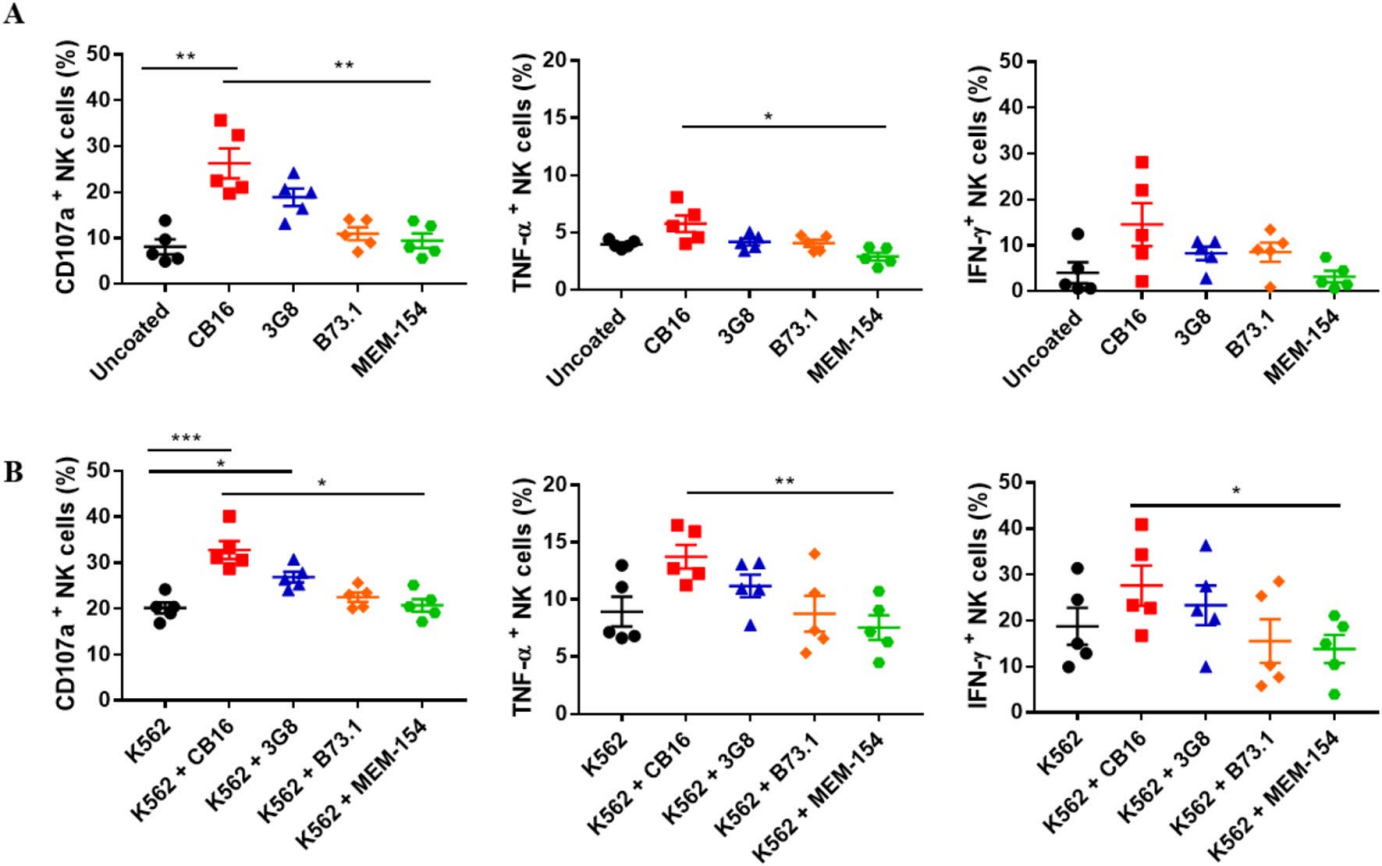
NK cell responses upon stimulation with different anti-CD16 antibody clones. CD107a, TNF-α and IFN-γ of NK cells in PBMCs from healthy donors were measured in the stimulation of (**A**) antibody alone or (**B**) costimulation of K562 and antibody. Antibody stimulations were induced by microbeads coated with various clones of CD16 antibodies, CB16, 3G8, B73.1 and MEM-154, respectively, for 6 h. All data are shown as the mean ± SEM (n = 5; *, *p* < 0.05; **, *p* < 0.01).

To compare the response of NK cells to the four anti-CD16 antibody clones, namely CB16, 3G8, B73.1, and MEM-154, we measured the amount of CD107a-, IFN-γ, and TNF-α positive NK cells in PBMCs after stimulation with anti-CD16 antibody-coated beads (Fig. 1A). NK cells stimulated with bead-coated CB16 clones displayed the highest expression of CD107a, TNF-α, and IFN-γ (not statistically) compared to those stimulated with uncoated beads. Beads coated with other clones, except the 3G8 clone in CD107a, did not significantly differ from the uncoated beads. When NK cells were costimulated with K562 and beads coated with anti-CD16 antibodies, only the CB16 clone showed further enhanced CD107a expression compared with K562, and the highest stimulating trends in TNF-α and IFN-γ were maintained (Fig. 1B).

### Proliferation capacity of NK cells and NKT cells upon stimulation with different anti-CD16 antibody clones

**Figure 2:**
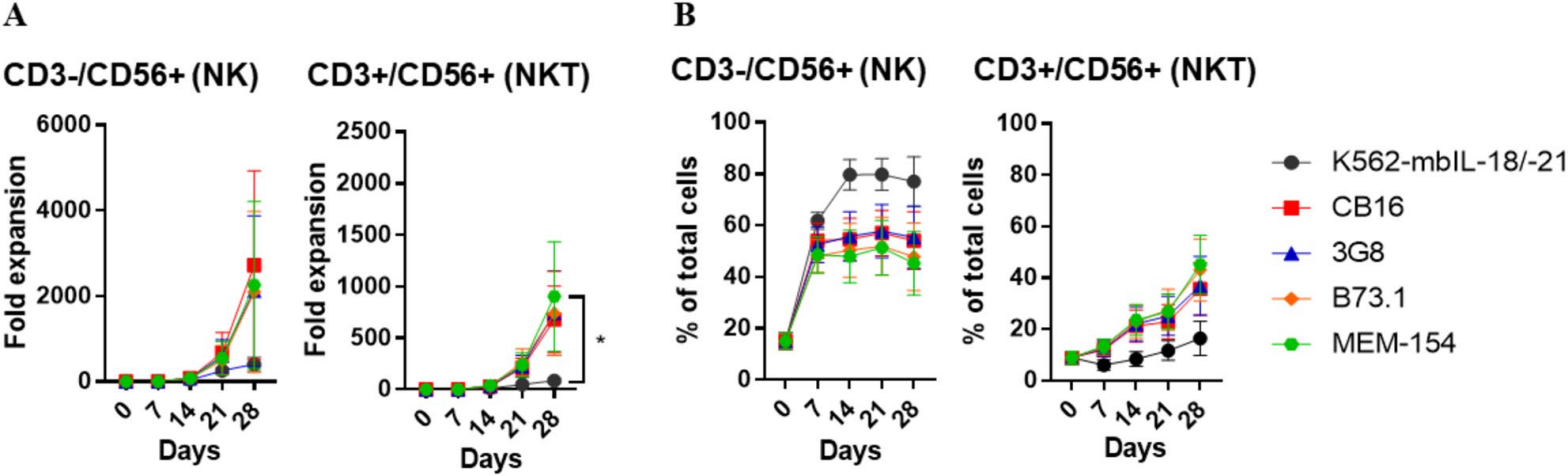
Proliferation capacity of NK and NKT cells upon stimulation with different anti-CD16 antibody clones. (**A**) Fold expansion of NK cells, CD3-/CD56+ and NKT cells, CD3+/CD56+, in the feeder cell, K562-mbIL-18/-21, or anti-CD16 antibody stimulation. (**B**) NK and NKT cells purity in the feeder cell, K562-mbIL-18/-21, or anti-CD16 antibody stimulation. Antibody stimulations were induced by microbeads coated with various clones of CD16 antibodies, CB16, 3G8, B73.1 and MEM-154, respectively. All data are shown as the mean ± SEM (n = 6; *, p < 0.05).

The proliferative capacity of NK cells and natural killer T (NKT) cells, known to express CD16 receptors among PBMCs, was examined after stimulation with different anti-CD16 antibody clones coated on beads or irradiated feeder cells (K562‑mbIL‑18/-21). For the preliminary study, PBMCs were isolated from blood stored in a cold storage leukoreduction system (LRS) chamber. NK cell (CD3-/CD56+) and NKT cell (CD3+/CD56+) purity (% of each group) and fold expansion were checked weekly until day 28. The fold expansion of NK cells and NKT cells was higher in all anti-CD16 antibody clones than in feeder cell stimulation (Fig. 2A). The purity of NK cells expanded by anti-CD16 antibody stimulation was lower than that of the feeder cells after day 14 (Fig. 2B). The decrease in NK cell purity is due to the outgrowth of NKT cells.

### Enhanced NK cell expansion via costimulation with K562‑mbIL‑18/-21 feeder cells and anti-CD16 antibodies

**Figure 3:**
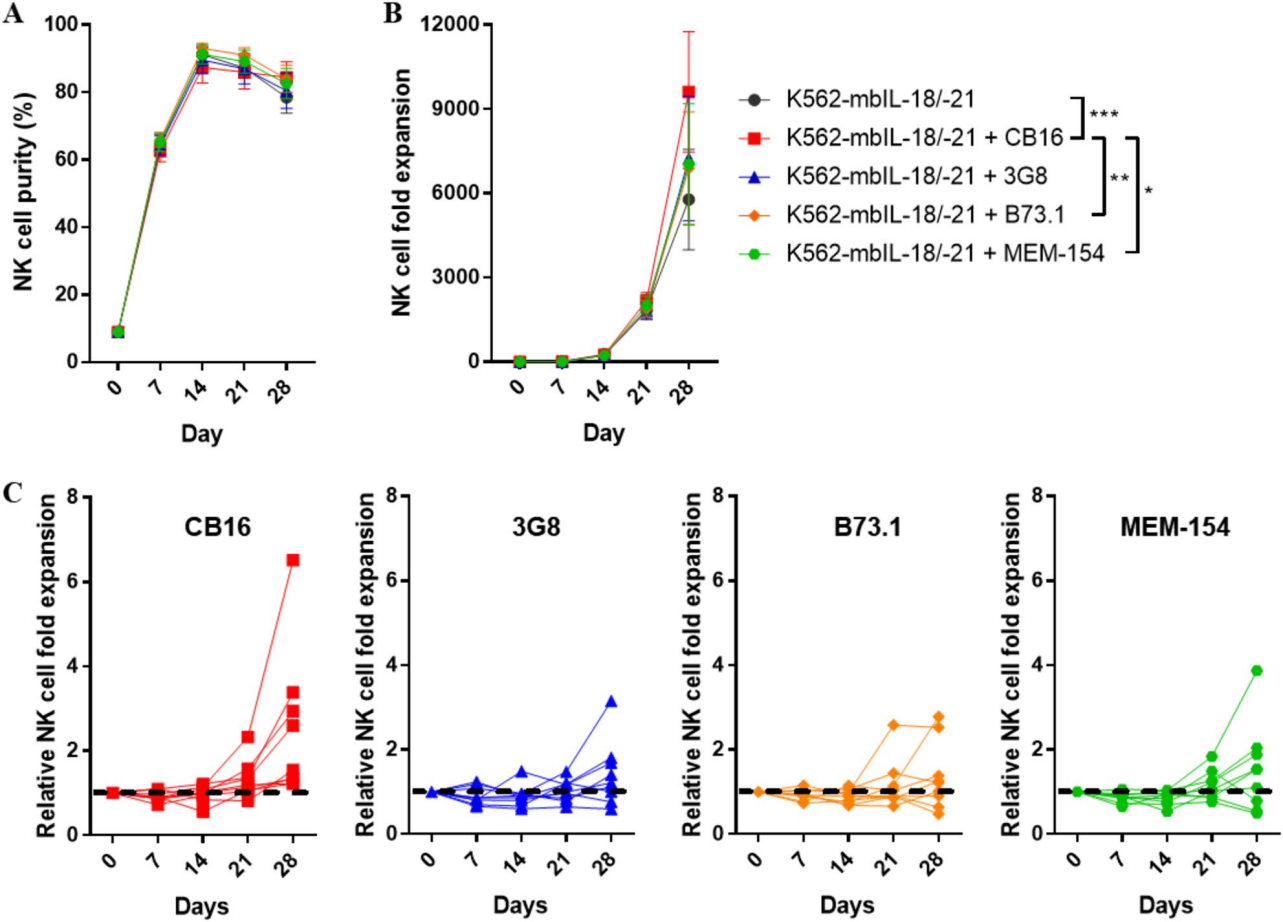
Enhanced NK cell expansion via costimulation with feeder cells and anti-CD16 antibodies. (**A**) NK cell purity and (**B**) fold expansion of NK cells in PBMCs cultured with feeder cell, K562-mbIL-18/-21, alone or costimulation with various clones of anti-CD16 antibodies, CB16, 3G8, B73.1 and MEM-154, coated on microbeads, respectively. (**C**) Relative NK cell fold expansion of each PBMC donor in four different costimulation condition. NK cell expansion in K562-mbIL-18/-21 alone was used a standard (dashed line). All data are shown as the mean ± SEM (n = 9; *, p < 0.05; **, p < 0.01; ***, p < 0.001).

Anti-CD16 stimulation was beneficial to NK and NKT cell proliferation, but was not sufficient to specifically stimulate NK cells when used alone. As a result, we combined anti-CD16 stimulation with the feeder cells, K562‑mbIL‑18/-21, which has superior specificity for NK cell proliferation. Thereafter, we used freshly prepared blood for NK cell expansion to estimate the maximum proliferation costimulation with K562-mbIL-18/-21 feeder cells and anti-CD16 antibodies coated on beads was found to result in a high level of NK cell purity (CD3-/CD56+), approximately 90%, after 14 days (Fig. 3A). All donors costimulated with the CB16 clone demonstrated a higher NK cell fold expansion compared to those stimulated with feeder cells alone. In contrast, several donors costimulated with other anti-CD16 clones (such as 3G8, B73.1, and MEM-154) exhibited a lower NK cell fold expansion by day 28 when compared to feeder cells alone stimulation (Fig. 3B,C).

### Effectiveness of the CB16 clone at stimulating NK cells

**Figure 4:**
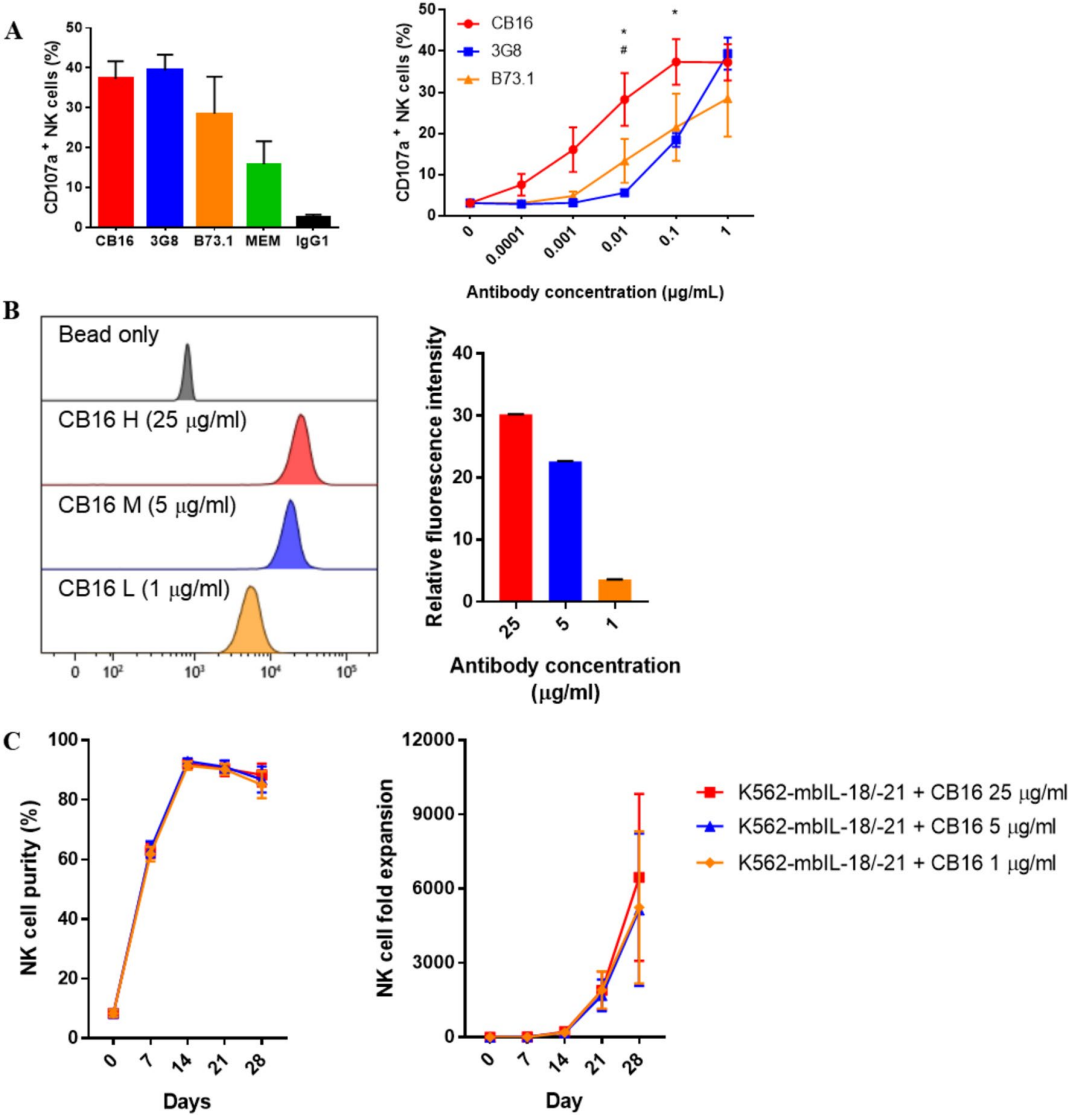
Effectiveness of the CB16 clone for NK cell stimulation. (**A**) CD107a expression on NK cell were measured after four anti-CD16 antibody clones (CB16, 3G8, B73.1, MEM-154) and negative control IgG1, all at a concentration of 1 μg/mL. CB16, 3G8, B73.1 were assessed at various antibody concentration for 6 h. (**B**) Microbeads coated with various concentration of CB16 clone were quantitatively measured by fluorescence labeled secondary antibodies. (**C**) NK cell purity and fold expansion of NK cells cultured in feeder cell, K562-mbIL-18/-21 alone or combination of microbeads coated with various concentration, 25, 5, 1 μg/mL, of CB16 clone. All data are shown as the mean ± SEM (n = 6 *#, p < 0.05, *CB16 vs B73.1; #CB16 vs 3G8).

NK cell stimulation measured via the CD107a degranulation assay revealed that CB16, 3G8, and B73.1 clones were found to induce stimulation of NK cells in high antibody concentration (1 μg/mL), and among them, the CB16 clone induced effective NK cell stimulation even at low antibody concentrations (Fig. 4A). Thus, we hypothesized that the CB16 clone could stimulate NK cells with a lower antibody coating density on the beads. We coated the beads with CB16 antibody at various coating densities (Fig. 4B). Surprisingly, NK cells costimulated with feeder cells and CB16 clones coated on beads at various concentrations did not show a meaningful difference in terms of NK cell fold expansion (Fig. 4C).

Most feeder cell-based NK cell expansion methods repeatedly treat feeder cells every week to maximize their expansion. As a result, we examined the restimulation effect of feeder cells by treating K562‑mbIL‑18/-21 cells 3 times at 7-day intervals. The CB16 clone restimulation condition could not be evaluated owing to the sudden death of NK cells after CB16 clone restimulation (data not shown). NK cell expansion was also enhanced by CB16 stimulation during feeder cell restimulation; restimulation led to a higher NK cell purity and fold expansion than single feeder cell stimulation on day 35 (Supplement Fig. 1).

### Characteristics of expanded NK cells costimulated with K562‑mbIL‑18/-21 feeder cell and CB16 clone

**Figure 5:**
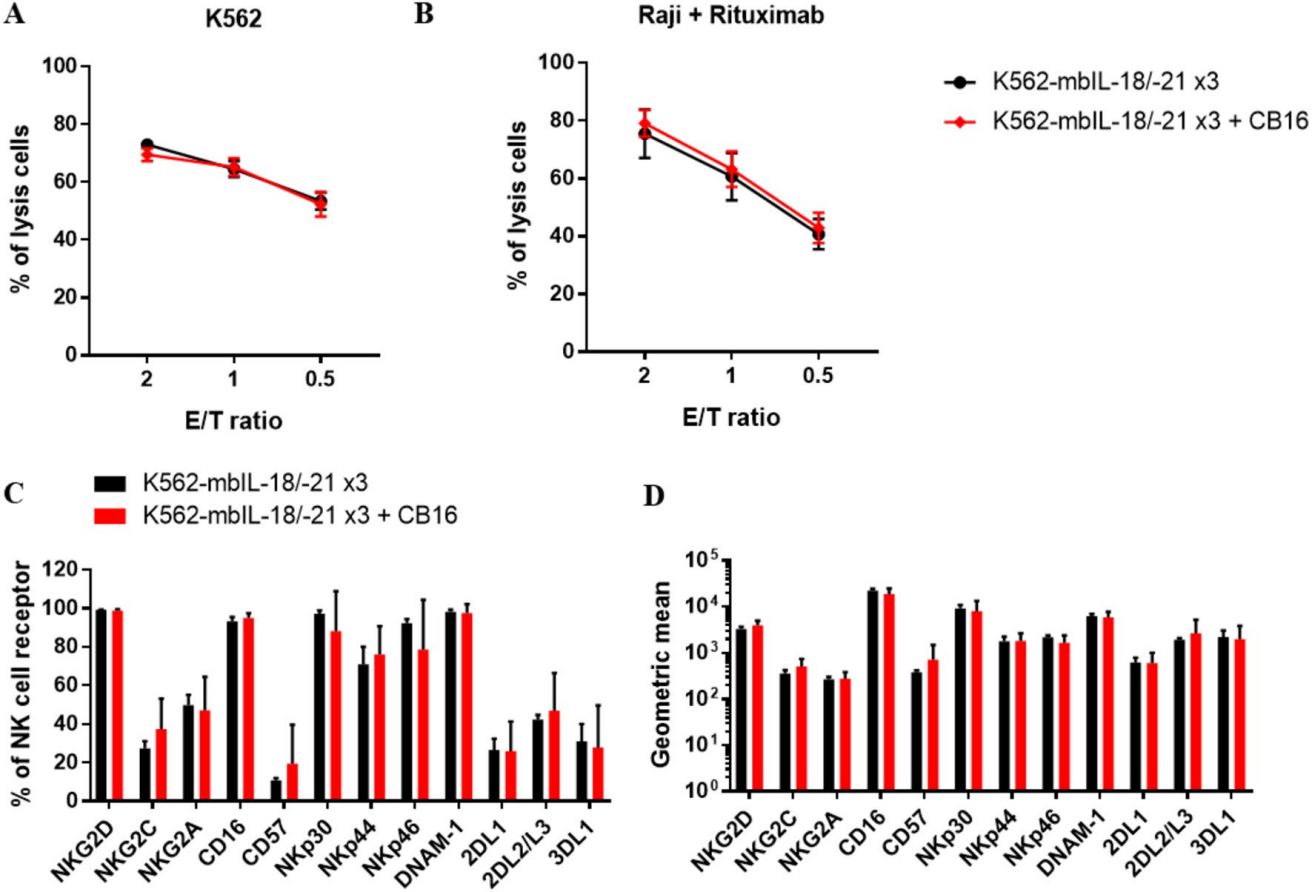
Characteristics of expanded NK cells costimulated with the feeder cell and CB16 clone. Cytotoxic activity of expanded NK against (**A**) K562 or (**B**) Raji coated with Rituximab cultured in repeated stimulation, day 0, 7 and 14, of feeder cells, K562-mbIL-18/-21, with or without CB16 coated on microbead for 28 days. (**C**) Percent expression levels and (**D**) geometric mean of NK cell surface receptors related to cytotoxic function after culture with indicated stimulation for 28 days. All data are shown as the mean ± SEM (n = 6).

To compare the cytotoxic function of NK cells expanded with feeder cell restimulation alone and costimulated with the CB16 clone, we measured the direct cytotoxicity and ADCC at day 28. No significant difference was found between NK cells expanded with feeder cell restimulation alone and costimulated with the CB16 clone at various E: T ratios (Fig. 5A). The expression levels of NK cell surface receptors (NKG2D, NKG2C, NKG2A, CD16, CD57, NKp30, NKp44, NKp46, DNAM-1, KIR2DL1, KIR2DL2/3, KIR3DL1) were similar to those of NK cells expanded with either K562‑mbIL‑18/-21 feeder cell restimulation alone or costimulated with the CB16 clone (Fig. 5B).

## Discussion

In the present study, we compared the effects of four clones of anti-CD16 antibodies on NK cell activation and expansion and revealed, for the first time, a higher performance of the CB16 clone. In addition, we demonstrated that the combination of an anti-CB16 clone coated on beads and the feeder cells, K562‑mbIL‑18/-21, markedly enhanced NK cell expansion.

Unlike other ITAM-coupled NK cell receptors, CD16 may independently activate dormant human NK cells, and this activation can be boosted by signals from other NK cell activation receptor^19^. A crucial transcription factor known as nuclear factor B (NF-κB) causes the reprogramming of gene transcription to produce cytokines and chemokines^20,21^. Upon identification of the target cell, NF-κB aids in activating the NK cell effector activities. Multiple activating receptor-ligand interactions, such as those involving NKG2D, 2B4, and DNAM-1, although activation of a single receptor is insufficient to for NF-κB to become active. According to a recent publication, the coactivation of 2B4 and NKG2D (or DNAM-1) results in a synergistic activation of the NF-κB by causing the stepwise phosphorylation of p65^21^. The generation of cytokines, natural cytotoxicity, and NK cell proliferation can all be dramatically induced by combinations of NK cell-activating receptors, including CD16. Anti-CD16 antibodies are reported to affect NK cell proliferation. Accordingly, the effect of four clones of anti-CD16 antibodies on NK cell expansion was evaluated. Several types of NK cell stimulation using antibodies targeting NK cell activating receptors, such as floating, plated bound, and coated on microbeads, have been reported. The floating antibody method, which involves the addition of antibodies to cell culture media, is a simple method; however, its stimulation strength and duration are limited^22,23^. Plated-bound forms of antibodies are the most common for antibody stimulation. Antibodies can be coated on certain types of cell culture plates, including surface-treated plates for adhesion cell culture, and incubated for several hours^17^. Thus, stimulated floating cells usually require untreated cell culture plates, which may trigger undesired responses. Microbeads coated with diverse biomolecules, including antibodies, can stimulate cells with more physiological signal strength by managing their size, similar to the physiological situation^24^. Moreover, relatively prolonged stimulation could be achieved using microbeads because they are not easily removed during cell culture. NK cells were reported to interact with microbeads coated with antibodies against activating receptors, and co-incubation of cultured NK cells with antibodies coated on microbeads also induced typical activating signaling events, resulting in induced NK cell function^25^. Based on these findings, a bead-coated anti-CD16 mAb was used to activate and expand NK cells.

Numerous monoclonal antibodies have been developed against CD16. In fact, anti-CD16 clones 3G8 and B73.1 have been commonly used to stimulate NK cells in previous studies^17,19,26–30^. Different functional responses and expansion of NK cells using different clones of anti-CD16 can be expected; however, systemic comparisons of the anti-CD16 antibody clones are limited. In this study, by measuring CD107a degranulation and IFN-γ and TNF-α cytokine production, we found that the NK cell response level differed among the four mAbs, and the CB16 clone was the most effective, followed by 3G8, B73.1, and MEM-154 (Fig. 1). This difference might be due to the distinct epitope recognition of each clone: the epitopes of CB16 and 3G8 are located on the putative FG loop of the membrane-proximal Ig-like domain (the major binding site for IgG), while the epitopes MEM-154 and B73.1 are shown to reside in the proximity of the binding site and the distal Ig domain of CD16^31–34^.

In this study, we first attempted to use the bead-coated anti-CD16 antibodies, CB16, 3G8, B73.1, and MEM-154, to expand NK cells. Thereafter, NK cells expanded using bead-coated anti-CD16 antibodies were compared with those expanded using K562-mbIL-18/-21 feeder cells. Although stimulation with the bead-coated anti-CD16 antibody led to a comparable number of NK cells (CD3-/CD56+) to be stimulated with K562‑mbIL‑18/‑21, stimulation with the bead-coated anti-CD16 antibody was not NK cell-specific (Fig. 2). Anti-CD16 antibodies also stimulate the NKT cell population (CD3+/CD56+), which has been reported to express the Fc receptor, CD16^35–37^. For selective NK cell expansion, anti-CD16 antibody stimulation was combined with feeder cells. All combinations of the CD16 clones led to high NK cell purities of over 90%; however, only the CB16 clone caused enhanced NK cell expansion for NK cells expanded with feeder cells only (Fig. 3). Our results suggest that the CB16 clone has the highest potential for NK cell expansion. Although we could not demonstrate it in this study, it is possible that the variation in NK cell expansion in response to CB16 clone stimulation among donors is associated with CD16 polymorphisms, similar to what is known for the binding of 3G8 clone to CD16^38^. Further research is required to investigate this hypothesis and determine whether CD16 polymorphisms are a significant factor influencing NK cell responses to CB16 clone stimulation. Moreover, the CB16 clone is effective for NK cell stimulation at lower concentrations; thus, it is not only cost-effective but can also be improved by adding other stimulating molecules to the beads (Fig. 4). The beads coated with the CB16 clone could not be further engineered for NK cell stimulation; 4-1BB agonist antibody or diverse ligands of NCR receptors may exhibit synergistic effects with the CB16 clone when coated on the same bead^39–41^.

Repeated treatment of feeder cells during NK cell expansion is a useful method to increase NK cell expansion, thus, many protocols use repeat stimulation^12,42^. NK cell stimulation with beads coated with the CB16 clone markedly increased NK cell expansion, despite three rounds of K562‑mbIL‑18/-21 restimulation (Supplement Fig. 1). Repeated stimulation with feeder cells combined with CB16 stimulation resulted in a slightly increased NK cell fold expansion and prominently higher NK cell purity. CB16 stimulation was beneficial for NK cell fold expansion, and feeder cell restimulation maintained high NK cell purity. NK cells expanded with the CB16 clone showed massive proliferation after day 21, which was maintained over day 35. Accordingly, stimulation of NK cells with the CB16 clone may induce long-term survival in vivo, which is an important feature of adoptively transferred NK cells^16,43,44^.

The characteristics of NK cells expanded using a combination of K562‑mbIL‑18/-21 cells with CB16 stimulation were compared to those of NK cells expanded with K562‑mbIL‑18/-21 cells. In addition to their enhanced expansion, NK cells grown with K562‑mbIL‑18/-21 cells with CB16 were similar to those grown with K562‑mbIL‑18/-21 cells based on various surface expression receptors, direct cytotoxicity, and ADCC (Fig. 5). Previous studies have shown that CD16 stimulation can enhance ADCC by increasing CD16 expression on the NK cell surface under physiological conditions^45^. However, in our study, NK cells expanded with K562‑mbIL‑18/-21 displayed high CD16 expression; thus, no difference in ADCC would be found upon CB16 stimulation.

In the current study, the CB16 clone of the anti-CD16 antibody was identified as the most effective clone for activating and proliferating NK cells. Moreover, promising NK cell expansion was achieved via costimulation with feeder cells and CB16 (Supplement Fig. 2). However, this method should be further characterized, such as bead elimination before harvesting NK cells, for clinical use. Overall, developing genetically engineered feeder cells to express the membrane-bound form of the CB16 clone might be better than using microbeads alone.

## Methods

### Cells and culture

K562 (human myelogenous leukemia cell line) and Raji (human Burkitt’s lymphoma cell line) cells were obtained from the American Type Culture Collection (ATCC, Manassas, VA, USA). The cells were cultured in RPMI 1640 medium supplemented with 10% heat-inactivated fetal bovine serum (FBS) (Gibco, US), 100 units/mL penicillin, and 100 μg/mL streptomycin (Invitrogen, CA, USA) at 37 °C in a humidified 5% CO_2_ incubator.

### Generation of genetically engineered K562 expressing mbIL-18/-21

GE-K562 cells were generated as described previously with slight modification^14^. 293FT cells were co-transfected with pCDH-CMV-mbIL-18/-21-EF1-RFP and a lentiviral packaging mixture (pLP1, pLP2, and pLP/VSVG; Thermo Scientific) to produce lenti-mbIL-18/-21. The transfections were performed using D-fectin (Lugen Sci Co. Ltd., Bucheon, South Korea) according to the manufacturer’s instructions. The K562 cells were transduced with lentiviral supernatant for 24 h in the presence of polybrene (8 μg/ml; Sigma-Aldrich, St. Louis, MO, USA) at 50 MOI (Multiplicity of Infection). Two weeks after transduction, GFP-positive cells were sorted using a BD FACSAria TM III and maintained in RPMI 1640 with 10% FBS.

### Bead coating

Beads were fabricated by modifying the surfaces of Dynabeads M-450 epoxy (1 ml; 4.0 × 10^8^ beads/ml; Thermo Fisher Scientific). Dynabeads were washed and resuspended in 1 ml buffer containing 0.1 M sodium phosphate and 1 M ammonium sulfate at pH 7.4. Streptavidin (40 ml; 5 mg/ml; Invitrogen) was added to the Dynabead suspension and incubated for 24 h at room temperature (RT) with gentle rotation for conjugation. The unreacted epoxy groups on the Dynabeads were quenched by adding 1 ml of Tris buffer (100 mM Tris; pH 7.9) to the reaction mixture and performing a 1 h incubation at RT. Streptavidin-coated Dynabeads were washed 3 times with PBS and resuspended in bicarbonate buffer (100 mM sodium bicarbonate; pH 8.3). One milliliter of bead was generated via mixing with biotinylated anti-CD16 on various clones: CB16 (Invitrogen), 3G8 (Biolegend), B73.1 (Invitrogen), MEM-154 (Invitrogen), for 10 min at RT, and then washing with PBS.

### Ex vivo NK cell expansion using K562‑mbIL‑18/-21 feeder cells with Bead coated CD16 antibody

Informed consent was obtained from all participants and the protocol was approved by the Institutional Review Board of the Samsung Medical Center, Seoul, Korea (IRB No. SMC 2021–09-111). All experiments were performed in accordance with relevant guidelines and regulations. Approved this study and no data were used for personal identification of human PBMCs. NK cells were expanded from PBMCs via co-culture with 100 Gy gamma-irradiated K562‑mbIL‑18/-21 cells, as described previously, with slight modifications^46,47^. Briefly, PBMCs were isolated from heparinized peripheral blood via density-gradient centrifugation with Ficoll-Hypaque (d = 1.077, Lymphoprep™; Axis-Shield, Oslo, Norway). PBMCs were co-cultured with irradiated K562‑mbIL‑18/-21 cells in a 24-well plate with RPMI 1640 medium (10% FBS, 100 U/mL penicillin, 100 µg/mL streptomycin, and 4 mmol/L L-glutamine) containing 10 U/mL recombinant human IL-2 with bead coated anti-CD16 antibody. After day 7, the concentration of IL-2 increased from 10 U/mL to 100 U/mL, and 5 ng/mL soluble IL-15 was added to the medium. The irradiated GE-K562 feeder cells were used for restimulation on days 7 and 14. The medium was replaced every 2–3 days. The expanded NK cells were cultured continuously until day 28. The expansion rate of NK cells is presented as the “expansion fold,” which was determined by dividing the absolute number of NK cells at the time points of interest by the respective number on day 0. The absolute number of NK cells was determined by gating CD56+/CD3- cells using fluorescence-activated cell sorting (FACS) and then counting the total number of cells.

### Cytokines and antibodies

Recombinant human IL-2 and IL-15 (PeproTech, Rocky Hill, NJ, USA) were used to expand NK cells from PBMCs. Allophycocyanin (APC)-Cy7-conjugated anti-human CD3, phycoerythrin (PE)-Cy7-conjugated anti-human CD56, PE-Cy5-conjugated CD56, and fluorescein isothiocyanate (FITC)-conjugated CD3 (eBioscience, San Diego, CA, USA) were used to measure NK cell purity (CD56+/CD3–). Pacific blue-conjugated anti-human CD16, FITC-conjugated anti-human CD57, APC-conjugated anti-human DNAM-1, peridinin chlorophyll protein complex (PerCP)-conjugated anti-human NKG2D, PE-conjugated anti-human NKG2C, APC-conjugated anti-human NKG2A, and PE-conjugated anti-human NKp30, PerCP-Cyanine5.5-conjugated anti-human NKp44, Pacific blue-conjugated anti-human NKp46 (eBioscience), PerCP-Cyanine5.5-conjugated anti-human KIR2DL1, FITC-conjugated anti-human KIR2DL2/3, and Pacific blue-conjugated anti-human KIR3DL1 (BD Biosciences, Franklin Lakes, NJ, USA) were used to measure expanded NK cell receptor levels. BV421-conjugated anti-human IFN-γ and APC-conjugated anti-human TNF-α (BD Biosciences) were used for intracellular staining, and PE-conjugated anti-human CD107a (BD Biosciences) was used as a surrogate marker for degranulation.

### Direct NK cytotoxicity assay

Expanded NK cells were harvested on day 28 to measure their cytotoxicity and ADCC against target tumor cells (K562 and Raji cells) by flow cytometry via carboxyfluorescein diacetate succinimidyl ester (CFSE; Life Technologies) staining of target cells, as previously described^13^. Briefly, K562 and Raji target cells were stained with 0.5 μM CFSE in fluorescence-activated cell sorting (FACS) buffer at 37 °C for 10 min and washed twice with RPMI medium. For the ADCC assay, Raji cells were stained with 0.5 μM CFSE in FACS buffer at 37 °C for 10 min and then washed twice with RC medium. CFSE-stained Raji cells were treated with 5 μg/mL rituximab. Target cells (2 × 10^4^) were transferred into a 96-well U-bottom plate in triplicate and mixed with various numbers of expanded NK cells (0.5:1, 1:1, and 2:1 effector-to-target ratios). The plates were centrifuged at 400 × g for 3 min and incubated for 4 h at 37 °C in a 5% CO_2_ incubator. After incubation, the co-incubated cells were transferred to FACS tubes. One microliter of 1 mg/mL propidium iodide (Sigma-Aldrich) was added to each tube prior to data acquisition. All functional assay data were acquired on a FACS Lyric instrument (BD Biosciences) and analyzed using Kaluza software version 2.1.

### NK activation assay

To analyze NK cell degranulation, PBMCs were used to assess the CD107a assay after 6 h of incubation at 37 °C and 5% CO_2_ in a humidified incubator. PBMCs were incubated with uncoated or coated bead anti-CD16 antibody (clone: CB16, 3G8, B73.1, MEM-154) in the absence or presence of K562 cells at an E:T ratio of 1:1 in a 96-well plate. Anti-CD107a was then added to capture CD107a as a marker of NK cell degranulation. Monensin and brefeldin A (BD Biosciences) were added after 1 h and incubated for another 5 h. To measure IFN-γ and TNF-α production, intracellular staining using the BD Cytofix/Cytoperm™ kit (BD Biosciences) was performed according to the manufacturer's instructions. After 5 h of incubation, the cells were harvested, washed with FACS buffer, and stained with FITC-conjugated anti-human CD3 and PE-Cy7-conjugated anti-human CD56 antibodies for 15 min on ice. The PBMCs were then washed, fixed, and permeabilized before being stained with BV421-conjugated anti-human IFN-γ and APC-conjugated anti-human TNF-α on ice for 30 min. Finally, the cells were washed and analyzed using FACS Lyric and Kaluza software.

### Statistical analysis

GraphPad Prism 7 was used to collect and analyze data. Statistical analyses of the differences between groups in terms of the purity, fold expansion, and cytotoxicity of expanded NK cells were carried out using the Friedman test, with a p-value of ≤ 0.05 considered to indicate significance.

## Supplementary Information

Supplementary Figures.

## Data Availability

All data produced or analyzed for this study are included in the published article and its supplemental information files. This study did not generate any code. Any additional information required to reanalyze the data reported in this paper is available from the lead contact on request.
