# Supplementary material for: Comparison of the different anti-CD16 antibody clones in the activation and expansion of peripheral blood NK cells
Source: Sci Rep. 2023 Jun 11;13:9493. doi: 10.1038/s41598-023-36200-6 (PMC10258201; doi:10.1038/s41598-023-36200-6)
Supplement: Supplementary file 1 — Supplementary Figures. [file 41598_2023_36200_MOESM1_ESM.docx]

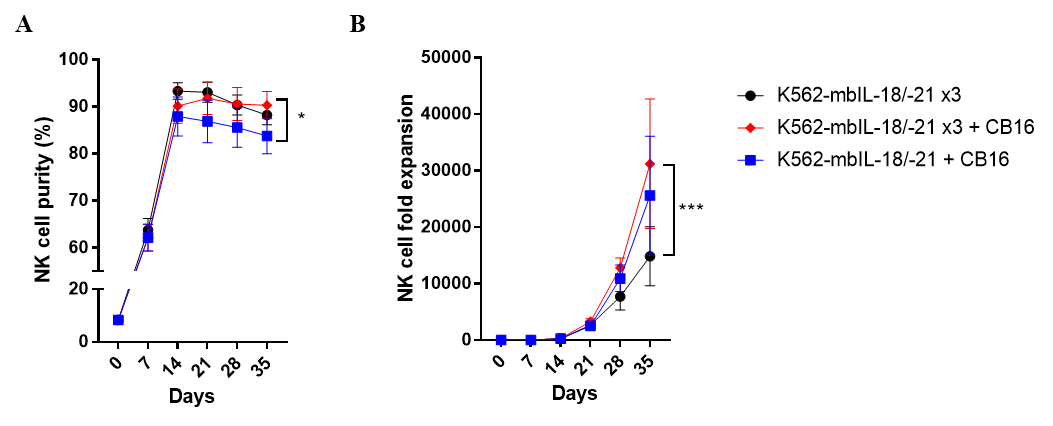


**Supplement Figure 1. Effect of feeder cell restimulation on NK cell expansion under CB16 co-stimulation.** (A) NK cell purity and (B) fold expansion of expanded NK cells cultured in repeated stimulation, day 0, 7 and 14, of feeder cells, K562-mbIL-18/-21, with or without CB16 coated on microbead for 35 days. All data are shown as the mean ± SEM (n = 10; *, p < 0.05; ***, p < 0.001).


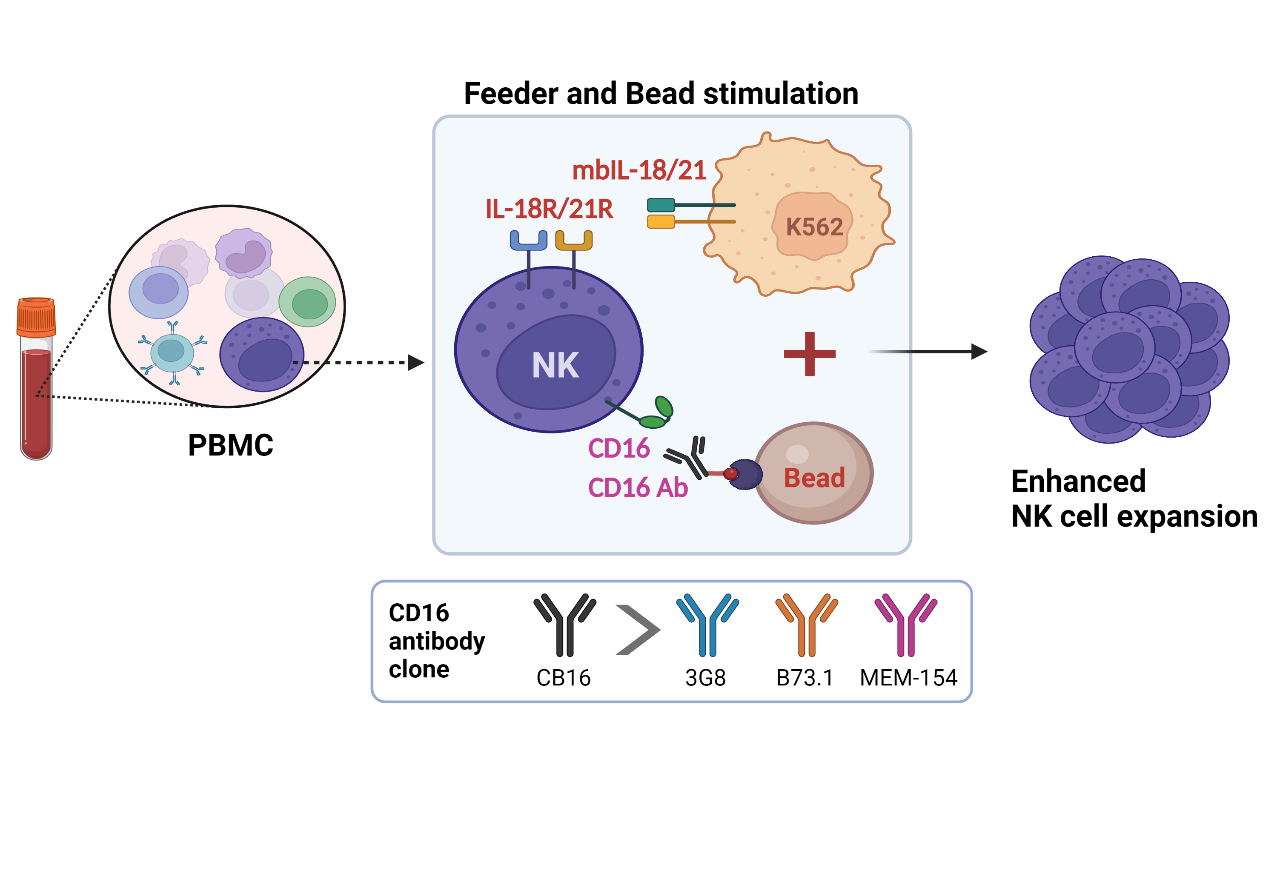
**Supplement Figure 2.** **Schematic illustrating a NK expansion method combination with anti-CD16 antibody coated bead and K562-mbIL-18/-21.**
